# Supplementary material for: Validity and Reliability of the COVID-19 Knowledge, Attitude and Behavior Scale
Source: Vaccines (Basel). 2023 Jan 31;11(2):317. doi: 10.3390/vaccines11020317 (PMC9960202; doi:10.3390/vaccines11020317)
Supplement: Supplementary file 1 [file vaccines-11-00317-s001.zip › vaccines-2105826-supplementary.pdf]

Supplementary File S1. CKAPS Draft.

Question	COVID 19 Knowledge-Attitude	Disagree	Partly agree	Totally agree
**K1**	COVID-19 disease transmitted by droplets in the coughs of patients.	1☐	2☐	3☐
**K2**	COVID-19 is more severe in the elderly and those with chronic diseases.	1☐	2☐	3☐
**K3**	The most common symptoms of COVID-19 disease are fever, cough and respiratory distress.	1☐	2☐	3☐
**K4**	There are methods that fully treat the COVID-19 disease.	1☐	2☐	3☐
**K5**	Mask should be worn to protect from COVID-19 disease.	1☐	2☐	3☐
**K6**	Hands should be washed with soap and water to protect from the virus.	1☐	2☐	3☐
**K7**	In the absence of water and soap, alcohol-containing disinfectant or cologne should be used.	1☐	2☐	3☐
**K8**	Contact of contaminated hands with eyes, mouth and nose may cause disease.	1☐	2☐	3☐
**K9**	Everyone who comes together to protect from disease must wear a mask.	1☐	2☐	3☐
**K10**	COVID-19 is not transmitted in areas with hot and humid climates.	1☐	2☐	3☐
**K11**	As social distancing decreases, the risk of transmission of COVID-19 disease increases.	1☐	2☐	3☐
**K12**	Those who have the flu vaccine are protected from COVID-19 disease.	1☐	2☐	3☐
**K13**	Washing the nose regularly with salty water protects the person from COVID-19 disease.	1☐	2☐	3☐
**K14**	Those who wear medical mask may come into close contact with people.	1☐	2☐	3☐
**K15**	The mask only needs to cover the mouth.	1☐	2☐	3☐
**K16**	Diluted sodium hypochlorite should be used for cleaning and disinfection of floors and surfaces.	1☐	2☐	3☐
**K17**	Those who have the pneumonia vaccine are protected from COVID-19 disease.	1☐	2☐	3☐
**K18**	Distance, hygiene and mask wearing rules should be followed among employees in order to protect themselves from disease in workplace.	1☐	2☐	3☐
**K19**	Natural ventilation should be preferred in homes and workplaces.	1☐	2☐	3☐
**K20**	The use of vinegar is effective in preventing COVID-19 disease.	1☐	2☐	3☐
**K21**	It is not necessary to wear a mask if there are a few people on public transport vehicles.	1☐	2☐	3☐
**K22**	Mask should be worn at home while in quarantine.	1☐	2☐	3☐
**K23**	Plants such as garlic and linden should be used to prevent COVID-19 disease.	1☐	2☐	3☐
**K24**	Used materials should not be reused after being removed.	1☐	2☐	3☐
**K25**	In case of fever, cough and shortness of breath, should go to the hospital immediately.	1☐	2☐	3☐
**K26**	The mask should be changed when it becomes damp or soiled.	1☐	2☐	3☐

Question	COVID 19 Knowledge-Attitude	Disagree	Partly agree	Totally agree
**K27**	Unnecessary and excessive use of disinfectants should be avoided.	1☐	2☐	3☐
**A1**	When a vaccine for COVID-19 disease is developed, I get it.	1☐	2☐	3☐
**A2**	Wearing gloves protects me from disease.	1☐	2☐	3☐
**A3**	If I wear a mask outside, I won’t get sick.	1☐	2☐	3☐
**A4**	COVID-19 is not easily transmitted to me.	1☐	2☐	3☐
**A5**	There is an unnecessary sensitivity in the society regarding COVID-19.	1☐	2☐	3☐
**A6**	COVID-19 is not that deadly disease is in my opinion.	1☐	2☐	3☐
**A7**	Even if I get COVID-19, I’ll get over it easily.	1☐	2☐	3☐
**A8**	I think my body resistance to COVID-19 disease is quite high.	1☐	2☐	3☐
**A9**	I do not believe that COVID-19 disease is transmitted by the scattering of droplets in the inhaled air.	1☐	2☐	3☐
**A10**	I do not believe that COVID-19 is transmitted by those who have had the disease asymptomatically.	1☐	2☐	3☐
**A11**	I do not believe that wearing a mask protects people from COVID-19 disease.	1☐	2☐	3☐
**A12**	I don’t think it is necessary to wash hands with soap and water to protect from COVID-19 disease.	1☐	2☐	3☐
**A13**	I think it is unnecessary to make a special effort to avoid getting COVID-19 disease.	1☐	2☐	3☐
**A14**	Social distancing doesn't matter to me in COVID-19 disease.	1☐	2☐	3☐
**A15**	I think that the COVID-19 disease will infect everyone.	1☐	2☐	3☐
**A16**	Only the elderly and chronic patients should be protected in the community for COVID-19 disease.	1☐	2☐	3☐
**A17**	Only sick people to wear mask is enough.	1☐	2☐	3☐
**A18**	Being in the same environment with COVID-19 patients is enough to catch the disease.	1☐	2☐	3☐
**A19**	I think it is important to wear disposable gloves to avoid contracting COVID-19.	1☐	2☐	3☐
**A20**	I think that the indoor environment should be ventilated frequently in order not to get COVID-19 disease.	1☐	2☐	3☐
**A21**	COVID-19 disease can cause more severe illness in smokers.	1☐	2☐	3☐
**A22**	Fans, air conditioners, hand-hair dryers can be important factors in the spread of COVID-19 disease in indoor environments.	1☐	2☐	3☐
**A23**	I do not believe that COVID-19 disease kills people without health problems.	1☐	2☐	3☐
**A24**	There is an excessive use of unnecessary disinfectants in society.	1☐	2☐	3☐
**A25**	I think the people around me are not taking the COVID-19 measures seriously enough.	1☐	2☐	3☐

Question	COVID 19 Behavior	Never	Sometimes	Often	Always
**B1**	I try to cover my mouth when I cough and sneeze.	1☐	2☐	3☐	4☐
**B2**	I keep a distance of at least 3-4 steps from people who show common cold symptoms.	1☐	2☐	3☐	4☐
**B3**	To protect myself from COVID-19 infection, I wear a mask when I go out.	1☐	2☐	3☐	4☐
**B4**	I use the mask to cover my mouth and nose.	1☐	2☐	3☐	4☐
**B5**	I wash my hands with soap and water, especially after touching surfaces that others have touched.	1☐	2☐	3☐	4☐
**B6**	In the absence of water and soap, I use alcohol-containing hand antiseptic or cologne.	1☐	2☐	3☐	4☐
**B7**	I do not touch the inside and outside of the mask while using it.	1☐	2☐	3☐	4☐
**B8**	I wear a mask in public transport vehicles.	1☐	2☐	3☐	4☐
**B9**	I take a break from visiting family and friends when illness is common.	1☐	2☐	3☐	4☐
**B10**	I act in accordance with social distance in the working and resting environment.	1☐	2☐	3☐	4☐
**B11**	I act in accordance with social distance in places where people eat collectively.	1☐	2☐	3☐	4☐
**B12**	I act in accordance with social distance in public transport vehicles.	1☐	2☐	3☐	4☐
**B13**	I wear a mask when I go out of the house when the disease is common.	1☐	2☐	3☐	4☐
**B14**	When the disease is common, when I come home from outside, I wash my hands with soap and water first.	1☐	2☐	3☐	4☐
**B15**	When the disease is widespread, I keep the shopping materials I brought home from outside for a while, then I process it.	1☐	2☐	3☐	4☐
**B16**	When the disease is common, I do not leave the house unless it is absolutely necessary.	1☐	2☐	3☐	4☐
**B17**	When illness is common, I try not to be in enclosed spaces where other people are present.	1☐	2☐	3☐	4☐
**B18**	I pay attention to how other people act outside.	1☐	2☐	3☐	4☐
**B19**	I pay attention to the social distance between me and other people when the disease is common.	1☐	2☐	3☐	4☐
**B20**	I am making a special effort to avoid contracting COVID-19.	1☐	2☐	3☐	4☐

Supplementary File S2. CKAPS.

**Knowledge-Contagion Dimension**	**Disagree**	**Partly agree**	**Totally agree**
KC1- COVID-19 disease is transmitted by droplets in the coughs of patients(K01).	1☐	2☐	3☐
KC2- COVID-19 is more severe in the elderly and those with chronic diseases(K02).	1☐	2☐	3☐
KC3- The most common symptoms of COVID-19 disease are fever, cough and respiratory distress(K03).	1☐	2☐	3☐
**Knowledge -Protection Dimension**			
KP1- Mask should be worn to protect from COVID-19 disease(K05).	1☐	2☐	3☐
KP2- Hands should be washed with soap and water to protect from the virus(K06).	1☐	2☐	3☐
KP3- In the absence of water and soap, alcohol-containing disinfectant or cologne should be used(K07).	1☐	2☐	3☐
KP4- Contact of contaminated hands with eyes, mouth and nose may cause disease(K08).	1☐	2☐	3☐
KP5- Everyone who comes together to protect from disease must wear a mask(K09).	1☐	2☐	3☐
KP6- Distance, hygiene and mask wearing rules should be followed among employees in order to protect themselves from disease in workplace(K18).	1☐	2☐	3☐
KP7- The mask should be changed when it becomes damp or soiled(K26).	1☐	2☐	3☐
**Attitude-Susceptibility Dimension**			
ASus1- I do not believe that COVID-19 disease is transmitted by the scattering of droplets in the inhaled air(A09).	1☐	2☐	3☐
ASus2- I do not believe that COVID-19 is transmitted by those who have had the disease asymptomatically(A10).	1☐	2☐	3☐
ASus3- I do not believe that wearing a mask protects people from COVID 19 disease(A11).	1☐	2☐	3☐
ASus4- I don't think it is necessary to wash hands with soap and water to protect from COVID 19 disease(A12).	1☐	2☐	3☐
**Attitude-Severity Dimension**			
Ase1- COVID-19 is not easily transmitted to me(A04).	1☐	2☐	3☐
Ase2- COVID-19 is not that deadly disease is in my opinion(A06).	1☐	2☐	3☐
Ase3- Even if I get COVID-19, I'll get over it easily(A07).	1☐	2☐	3☐
Ase4- think my body resistance to COVID-19 disease is quite high(A08).	1☐	2☐	3☐
**Attitude-Benefit Dimension**			
ABen1- Wearing gloves protects me from disease(A02).	1☐	2☐	3☐
ABen2- I won't get sick if I wear a mask outside(A03).	1☐	2☐	3☐
**Attitude-Barrier Dimension**			
ABar1- Social distance does not matter to me in COVID-19 disease(A14).	1☐	2☐	3☐
ABar2- Only the elderly and chronic patients should be protected in the community for COVID-19 disease(T16).	1☐	2☐	3☐
ABar3- Only sick people to wear mask is enough(T17).	1☐	2☐	3☐

Behavior-SeekingHealth Behavior Dimension	Never	Sometimes	Often	Always
BS1- I try to cover my mouth when I cough and sneeze(B01).	1☐	2☐	3☐	4☐
BS2- To protect myself from COVID 19 infection, I wear a mask when I go out(B03).	1☐	2☐	3☐	4☐
BS3- I use the mask to cover my mouth and nose(B04).	1☐	2☐	3☐	4☐
BS4- In the absence of water and soap, I use alcohol-containing hand antiseptic or cologne(B06).	1☐	2☐	3☐	4☐
BS5- I wear a mask in public transport vehicles(B08).	1☐	2☐	3☐	4☐
BS6- When the disease is common, when I come home from outside, I wash my hands with soap and water first(B14).	1☐	2☐	3☐	4☐
**Behavior-Avoidance Illness Dimension**				
BA1- I take a break from visiting family and friends when illness is common(B09).	1☐	2☐	3☐	4☐
BA2- I act in accordance with social distance in the working and resting environment(B10).	1☐	2☐	3☐	4☐
BA3- In public transport, I act in accordance with social distancing(B12).	1☐	2☐	3☐	4☐
BA4- When illness is common, I try not to be in enclosed spaces where other people are present(B17).	1☐	2☐	3☐	4☐
BA5- I pay attention to how other people act outside(B18).	1☐	2☐	3☐	4☐
BA6- I pay attention to the social distance between me and other people when the disease is common(B19).	1☐	2☐	3☐	4☐
